# Supplementary material for: Association between estimated pulse wave velocity and stroke in middle-aged and elderly Chinese: A nationwide cohort study
Source: PLoS One. 2025 Nov 21;20(11):e0336247. doi: 10.1371/journal.pone.0336247 (PMC12637936; doi:10.1371/journal.pone.0336247)
Supplement: Attachment — Submitted filename: Response to Reviewers.docx [file pone.0336247.s003.docx]

21 April, 2025

Michel Goldman

Editor-in-Chief

**PLOS ONE**

Dear Editor,

Thank you so much for your e-mail dated 10 April 2024, providing yours and the Reviewers’ comments on our submission (Manuscript ID: PONE-D-25-06790) entitled “Association between estimated pulse wave velocity and stroke in middle-aged and elderly Chinese: a nationwide cohort study”. Following your suggestions and the reviewers` comments, our manuscript has been revised completely and re-submitted, along with the point-by-point responses to reviewers.

In the re-submitted manuscript, the revised parts have been marked in yellow. We believe that the revised manuscript can meet the publication standards of your journal.

Thank you again for your help in improving our manuscript.

Yours sincerely,

Shengren Xiong

Department of Orthopaedics

Fujian Medical University Union Hospital

Fujian Medical University

No.29 Xinquan Road

Fuzhou 350001

Fujian Province

P.R. of China

Phone: +86 15859109188

E-mail: Xsrenzj@163.com

**Reviewer 1 comments:**

*Q1:* *The introduction effectively establishes the research gap by highlighting the limited studies on the relationship between ePWV and stroke risk, particularly in Asian populations. However, the novelty of the study could be further emphasised by contrasting it more explicitly with prior research. A more detailed comparison of their findings and methodologies would strengthen the justification for this study.*

**Response to the comment:** Thank you so much for your positive and constructive suggestions. Following your suggestions, the explicit comparison with previous studies has been provided to strengthen the justification in the introduction section. Please see lines 65-167.

(Lines 65-67) Most existing studies are focused on western populations [12,14], while fewer studies have been conducted in Asian populations (especially Chinese populations).

*Q2: The statistical analysis is robust, with appropriate adjustments for demographic, socioeconomic, and cardiometabolic factors. However, the rationale for selecting specific covariates, such as smoking, alcohol consumption, BMI, etc., could be better explained. Are these factors known to confound the relationship between ePWV and stroke risk? A brief justification would enhance transparency.*

**Response to the comment:** Thank you for your valuable comments. Yes, based on previous studies, smoking, alcohol consumption, and BMI were identified as confounders in the relationship between ePWV and stroke risk. A brief justification has been provided in the methods section to enhance transparency. Please see lines 113-115.

(Lines 113-115) Smoking, alcohol consumption, and BMI were selected as potential confounders in the relationship between ePWV and stroke risk based on previous studies [18,19].

*Q3:* *The use of restricted cubic spline analysis to explore the dose-response relationship is commendable. However, the choice of knots (10th, 50th, and 90th percentiles) should be justified. Were these percentiles chosen based on prior literature or exploratory analysis?*

**Response to the comment:** Thank you for your comments. Yes, the choice of knots (10th, 50th, and 90th percentiles) was based on prior literature.

*Q4: The description of the CHARLS cohort is clear, but more details on the sample's representativeness would be helpful. For example, how does the demographic profile of the study participants compare to the general Chinese population?*

**Response to the comment:** Thank you so much for your positive and constructive comments. Following your suggestions, the details on the sample's representativeness have been provided in the methods section. Additionally, details of the study design have already been reported. Please see lines 74-78.

(Lines 74-78) The study participants were middle-aged and elderly adults from CHARLS, an ongoing, nationally representative, population-based, prospective, longitudinal study (http://charls.pku.edu.cn/). The primary aim of this study was to recruit participants aged 45 years and above, but some participants aged 40 to 44 years also attended the baseline survey. Details of the study design have already been reported [15].

*Q5: The study acknowledges its limitations, including the lack of racial and regional diversity and potential selection bias due to missing data. However, the discussion could delve deeper into how these limitations might affect the interpretation of the results. For example, how might the findings differ in populations with different genetic or environmental risk factors?*

**Response to the comment:** Thank you so much for your valuable suggestions. Following your suggestions, we have expanded the Discussion to address how genetic, and environmental differences could influence the observed associations. Please see lines 228-236.

(Lines 228-236) First, the generalizability of our findings may be limited by the lack of racial and regional diversity in the study sample. As the CHARLS cohort consists exclusively of middle-aged and older Chinese adults, our results may not fully reflect the association between ePWV and stroke risk in populations with different genetic backgrounds, lifestyles, or environmental exposures. For instance, genetic predispositions affecting vascular function, as well as differences in dietary patterns, air pollution exposure, and healthcare access across regions or ethnicities, may modify the strength or direction of the observed associations. Further validation is needed in other racial and national populations.

*Q6: The exclusion of stroke subtypes (ischemic vs. hemorrhagic) is a significant limitation. The authors should elaborate on why this differentiation was not made and how it might impact the clinical applicability of the findings.*

**Response to the comment:** Thank you for your constructive suggestions. Yes, a limitation of our study is the the exclusion of stroke subtypes (ischemic vs. hemorrhagic), which has been addressed in the limitation section. Due to the unavailability of stroke subtype information in CHARLS, we were unable to differentiate between ischemic and hemorrhagic stroke. Our inability to differentiate between them may limit the clinical applicability of our findings. Future studies should explore the relationship between ePWV and different stroke subtypes. Please see lines 239-242.

(Lines 239-242) Lastly, due to the unavailability of stroke subtype information in CHARLS, we were unable to differentiate between ischemic and hemorrhagic stroke. Future studies should explore the relationship between ePWV and different stroke subtypes.

*Q7: The conclusion could be strengthened by briefly reiterating the clinical implications and suggesting directions for future research, such as investigating ePWV in diverse populations or exploring its relationship with stroke subtypes.*

**Response to the comment:** Thank you for your constructive suggestions. We have revised the Conclusion to include these points. Specifically, we highlight that ePWV, as a non-invasive surrogate of arterial stiffness, could serve as a practical tool for stroke risk stratification in middle-aged and older adults. We also recommend further research in diverse ethnic groups and analyses of specific stroke subtypes. Please see lines 246-252.

(Lines 246-252) As a simple and non-invasive surrogate marker of arterial stiffness, ePWV may serve as a useful tool for early identification of individuals at high risk of stroke in clinical settings. Future research should investigate this association in more diverse populations and explore the relationship between ePWV and specific stroke subtypes, such as ischemic and hemorrhagic stroke, to enhance the clinical applicability of ePWV in stroke prevention strategies.

**Reviewer 2 Comments:**

*Q1:* *Under Table 1. Characteristics of study participants according to ePWV. Variables "rural" and "Illiteracy", the numbers are quite high 6141(66.82%) and 4358(47.42%) respectively. it would be insightful if authors can give a brief statement in relation to occurrence of stroke in the Chinese population.*

**Response to the comment:** Thank you for your kindly reminding. Following your suggestions, we have included a brief statement regarding the occurrence of stroke in the revised manuscript and presented it in Table 1. Please see lines 145-147.

(Lines 145-147) During the follow-up, 678 participants (7.38 %) experienced a stroke. Participants in the lowest ePWV tertile were more likely to be non-smokers and non-drinkers, have a normal BMI, and be free of hypertension, diabetes, dyslipidemia and stroke (P < 0.05).

**Table 1. Characteristics of study participants according to ePWV**

| **Variables** | **Total** | **ePWV** | | | **P value** |
| --- | --- | --- | --- | --- | --- |
|  |  | **T1** | **T2** | **T3** |  |
| No. of participants | 9190 | 3064 | 3063 | 3063 |  |
| Age, Mean ± SD | 58.28 ± 8.75 | 51.82 ± 5.12 | 57.02 ± 6.03 | 66.00 ± 8.01 | <0.001 |
| Sex, n(%) |  |  |  |  | <0.001 |
| Male | 4227 (46.00) | 1248 (40.73) | 1431 (46.72) | 1548 (50.54) |  |
| Female | 4963 (54.00) | 1816 (59.27) | 1632 (53.28) | 1515 (49.46) |  |
| Residence, n(%) |  |  |  |  | 0.458 |
| Rural | 6141 (66.82) | 2066 (67.43) | 2054 (67.06) | 2021 (65.98) |  |
| Urban | 3049 (33.18) | 998 (32.57) | 1009 (32.94) | 1042 (34.02) |  |
| Education Level, n(%) |  |  |  |  | <0.001 |
| Illiteracy | 4358 (47.42) | 1246 (40.67) | 1425 (46.52) | 1687 (55.08) |  |
| Primary school | 2032 (22.11) | 615 (20.07) | 670 (21.87) | 747 (24.39) |  |
| Middle school | 1865 (20.29) | 804 (26.24) | 653 (21.32) | 408 (13.32) |  |
| High school or above | 935 (10.17) | 399 (13.02) | 315 (10.28) | 221 (7.22) |  |
| Marital status, n(%) |  |  |  |  | <0.001 |
| Married | 991 (10.78) | 158 (5.16) | 229 (7.48) | 604 (19.72) |  |
| Others | 8199 (89.22) | 2906 (94.84) | 2834 (92.52) | 2459 (80.28) |  |
| Smoking status, n(%) |  |  |  |  | <0.001 |
| No | 5643 (61.40) | 1999 (65.24) | 1874 (61.18) | 1770 (57.79) |  |
| Yes | 3547 (38.60) | 1065 (34.76) | 1189 (38.82) | 1293 (42.21) |  |
| Alcoholing status, n(%) |  |  |  |  | <0.001 |
| No | 5657 (61.59) | 1975 (64.52) | 1872 (61.12) | 1810 (59.13) |  |
| Yes | 3528 (38.41) | 1086 (35.48) | 1191 (38.88) | 1251 (40.87) |  |
| BMI, n(%) |  |  |  |  | <0.001 |
| Underweight | 581 (6.46) | 171 (5.69) | 156 (5.20) | 254 (8.51) |  |
| Normal | 5610 (62.37) | 2001 (66.57) | 1809 (60.26) | 1800 (60.28) |  |
| Overweight | 2346 (26.08) | 726 (24.15) | 866 (28.85) | 754 (25.25) |  |
| Obesity | 457 (5.08) | 108 (3.59) | 171 (5.70) | 178 (5.96) |  |
| Hypertension, n(%) | 3610 (39.28) | 549 (17.92) | 1194 (38.98) | 1867 (60.95) | <0.001 |
| Diabetes, n(%) | 1121 (12.21) | 301 (9.83) | 408 (13.35) | 412 (13.46) | <0.001 |
| Dyslipidemia, n(%) | 1572 (17.18) | 415 (13.61) | 566 (18.60) | 591 (19.33) | <0.001 |
| Stroke, n(%) | 678 (7.38) | 128 (4.18) | 237 (7.74) | 313 (10.22) | <0.001 |

ePWV: Estimated pulse wave velocity, SD: standard deviation

*Q2: Figure 1. Flowchart of study participant inclusion. Improve it by matching what has been written under the section "Study population and design." The sentence; "Had missing age information". If it can be added to the sentence that reads - "777 participants aged < 45" as it has been stated in text "we excluded individuals*

*who were under 45 years of age or had* *missing age information (n = 777)".*

**Response to the comment:** Thank you for your valuable comments. Following your suggestions, the "777 participants aged < 45" has been corrected in the Figure 1. Please see Figure 1.

****Fig 1.** Flowchart of study participant inclusion.**

*Q3: Authors mention that "Had incomplete follow-up data." but the flowcharts states that "3595 participants who were lost during follow up." If authors can relook at this and make it uniform for clarity.*

**Response to the comment:** Thank you for your constructive suggestions. Following your suggestions, the "Had incomplete follow-up data" has been corrected for clarity and consistency in the revised manuscript. Please see lines 87-90.

(Lines 87-90) Among the 17,708 baseline participants, we excluded individuals who were under 45 years of age or had missing age information (n = 777), lacked ePWV data (n = 3,689), lacked stroke data (n = 46), had a history of stroke or cardiovascular disease at baseline (n = 411), or were lost to follow-up (n = 3,595).

*Q3: All relevant data arewithin the paper and its Supporting Information files. Add space between "are" and "within".*

**Response to the comment:** Thank you for your valuable comments. Following your suggestions, the misspelled term "arewithin" has been corrected in the revised manuscript. Please see lines 268.

(Lines 268) All relevant data are within the paper and its Supporting Information files.
